# Sleep disturbances and the speed of multimorbidity development in old age: results from a longitudinal population-based study

**DOI:** 10.1186/s12916-020-01846-w

**Published:** 2020-12-07

**Authors:** Shireen Sindi, Laura Monica Pérez, Davide L. Vetrano, Federico Triolo, Ingemar Kåreholt, Linnea Sjöberg, Alexander Darin-Mattsson, Miia Kivipelto, Marco Inzitari, Amaia Calderón-Larrañaga

**Affiliations:** 1 Division of Clinical Geriatrics, Center for Alzheimer Research, Karolinska Institutet, Stockholm, Sweden; 2 Neuroepidemiology and Ageing Research Unit, School of Public Health, Imperial College London, London, UK; 3 Aging Research Center, Department of Neurobiology, Care Sciences and Society, Karolinska Institutet and Stockholm University, Stockholm, Sweden; 4 REFiT Barcelona Research Group, Vall d’Hebrón Research Institute and Parc Sanitari Pere Virgili, Barcelona, Spain; 5 Centro di Medicina dell’Invecchiamento, IRCCS Fondazione Policlinico “A. Gemelli” and Catholic University of Rome, Rome, Italy; 6 Institute of Gerontology, School of Health and Welfare, Aging Research Network – Jönköping (ARN-J), Jönköping University, Jönköping, Sweden; 7 Institute of Public Health and Clinical Nutrition and Institute of Clinical Medicine, Neurology, University of Eastern Finland, Kuopio, Finland; 8 Theme Aging, Karolinska University Hospital, Stockholm, Sweden; 9 Research and Development Unit, Stockholms Sjukhem, Stockholm, Sweden; 10 Department of Medicine, Universitat Autònoma de Barcelona, Barcelona, Spain

**Keywords:** Sleep disturbances, Multimorbidity, Aging, Cardiovascular, Neuropsychiatric, Musculoskeletal

## Abstract

**Background:**

Sleep disturbances are prevalent among older adults and are associated with various individual diseases. The aim of this study was to investigate whether sleep disturbances are associated with the speed of multimorbidity development among older adults.

**Methods:**

Data were gathered from the Swedish National study of Aging and Care in Kungsholmen (SNAC-K), an ongoing population-based study of subjects aged 60+ (*N* = 3363). The study included a subsample (*n* = 1189) without multimorbidity at baseline (< 2 chronic diseases). Baseline sleep disturbances were derived from the Comprehensive Psychiatric Rating Scale and categorized as none, mild, and moderate–severe. The number of chronic conditions throughout the 9-year follow-up was obtained from clinical examinations. Linear mixed models were used to study the association between sleep disturbances and the speed of chronic disease accumulation, adjusting for sex, age, education, physical activity, smoking, alcohol consumption, depression, pain, and psychotropic drug use. We repeated the analyses including only cardiovascular, neuropsychiatric, or musculoskeletal diseases as the outcome.

**Results:**

Moderate–severe sleep disturbances were associated with a higher speed of chronic disease accumulation (*ß*/year = 0.142, *p* = 0.008), regardless of potential confounders. Significant positive associations were also found between moderate–severe sleep disturbances and neuropsychiatric (*ß*/year = 0.041, *p* = 0.016) and musculoskeletal (*ß*/year = 0.038, *p* = 0.025) disease accumulation, but not with cardiovascular diseases. Results remained stable when participants with baseline dementia, cognitive impairment, or depression were excluded.

**Conclusion:**

The finding that sleep disturbances are associated with faster chronic disease accumulation points towards the importance of early detection and treatment of sleep disturbances as a possible strategy to reduce chronic multimorbidity among older adults.

**Supplementary Information:**

The online version contains supplementary material available at 10.1186/s12916-020-01846-w.

## Background

Sleep disturbances are a “public health problem,” as a third of adults report having insufficient sleep [1, 2], and half of older adults report some type of sleep disturbance [3]. While the prevalence of diagnosed insomnia disorder ranges from 5 to 8%, 20–40% report insomnia, and up to 50% complain of sleep problems [4, 5]. It has been estimated that the mean total healthcare costs were 75% higher for groups with moderate and severe insomnia compared to those with no insomnia, of a health plan sample in the USA [6]. Similarly, those with untreated insomnia have higher all-cause healthcare utilization [7], providing evidence for the economic burdens of (undiagnosed/untreated) sleep disturbances.

Chronic sleep disturbances have a range of negative health consequences, including hypertension, cardiovascular diseases, stroke, obesity, diabetes, migraines, and mortality [8, 9]. Sleep disturbances are also associated with an increased risk for dementia [10, 11], cognitive impairment [12–14], and depressive symptoms [15, 16]. Another important consequence of sleep disturbances is daytime sleepiness/drowsiness, which is associated with falls, death, and accidents [17, 18]. In a National Sleep Foundation survey, the frequency of sleep problem reports among older adults was 53% among those with no medical condition and escalated up to 80% among those with 4+ medical conditions, demonstrating the need for further investigations [18, 19]. Similarly, insomnia was associated with multiple medical and psychiatric comorbidities in a large analysis of Medicare beneficiaries (USA) [7].

To understand the broad implications of chronic sleep disturbances, it is important to simultaneously investigate the associations between sleep disturbances and multiple health outcomes. *Multimorbidity* refers to the coexistence of two or more chronic medical conditions in an individual [20]. Although multimorbidity is the norm among older adults [21, 22], there are inter-individual differences in the speed of multimorbidity development and progression.

Cross-sectional studies showed that short sleep duration, feelings of drowsiness, and difficulties sleeping were associated with multimorbidity [23, 24]. In a multi-national cross-sectional study, a linear dose-dependent association was found between the number of chronic conditions and sleep disturbances [25]. Findings from a US middle-aged sample showed that among several health-promoting behaviors, only sleep and physical activity were associated with multimorbidity [26]. Sex differences have also been demonstrated, including differences in the relationship between sleep disturbances and commonly co-occurring chronic conditions [27]. The association between sleep disturbances and multimorbidity may be bidirectional. For example, one study showed that the number of physical conditions at baseline was associated with incident insomnia two years later [28].

The aim of this study was to investigate the association between sleep disturbances and the speed of multimorbidity development, a proxy for loss of resilience and multisystem homeostatic dysregulation in older adults [29, 30].

## Methods

### Study design and population

The study population was derived from the Swedish National study of Aging and Care in Kungsholmen (SNAC-K), an ongoing population-based study, which enrolled adults aged 60 years and older living in the community or in institutions in the Kungsholmen district of Stockholm between 2001 and 2004 [31]. A total of 4590 people from 11 age cohorts (60, 66, 72, 78, 81, 84, 87, 90, 93, 96, and ≥ 99 years) were randomly invited to participate in the study. Finally, 3363 accepted to participate in the baseline examination (73.3% participation rate). Follow-up assessments are performed every six years for younger participants (60–78 years) and every three years for older participants (≥ 78 years). In the current study, we included a subsample of 1189 participants without multimorbidity at baseline (i.e., < 2 chronic disease) and with data for the main exposure.

SNAC-K participants undergo a comprehensive clinical and functional assessment by trained personnel at baseline and follow-ups. Data on past medical history and vital status were also available through the National Patient Register and the Swedish Death Register.

### Ethics, consent, and permissions

SNAC-K was approved by the Regional Ethical Review Board in Stockholm in Sweden. Written informed consent was collected from all participants or, if the participant was not capable of providing such consent, it was obtained from a proxy.

### Multimorbidity assessment

The total number of chronic conditions at baseline and at the follow-ups was operationalized according to the comprehensive list proposed by Calderon-Larrañaga et al., described elsewhere [32]. All the diagnoses were coded according to the International Classification of Diseases, 10th revision (ICD-10) and then classified as chronic or not chronic; then, those classified as chronic were grouped into 60 broader disease categories. Clinical information, laboratory test results, current treatment, and data from primary care and hospital medical records were also used to identify specific chronic conditions. Chronic conditions considered cardiovascular risk factors (i.e., hypertension, dyslipidemia, and obesity) were excluded from the total count consistent with previous studies [33]. Sleep disorders (ICD-10 codes F510-13 and G47) were also excluded to avoid exposure–outcome circularity.

### Sleep disturbances

The presence of sleep disturbances at baseline was assessed by physicians through the Comprehensive Psychiatric Rating Scale (CPRS), a scale rating the severity of psychiatric symptoms and observed behavior and change in symptoms over time [34]. The degree of sleep disturbances was categorized into three groups according to participants’ answers to the CPRS items on sleep: no sleep disturbances, mild sleep disturbances (grouping the categories 1–2 “Slight difficulty dropping off to sleep or slightly reduced, light or fitful sleep”), and moderate–severe sleep disturbances (grouping the categories 3–4 “Sleep reduced or broken by at least two hours” and 5–6 “Less than two or three hours of sleep”).

### Covariates

Education was measured as the highest level of formal education and was categorized as elementary school, high school, and university or above. Physical activity was categorized into three groups of intensity: inadequate, health-enhancing, and fitness-enhancing [35]. Smoking habit was categorized as never, former, or current smoker, and alcohol consumption as never, light/moderate, and heavy consumption. Body mass index (BMI) was calculated by dividing weight in kilograms by the square of height in meters and then categorized as underweight (< 18.5 kg/m^2^), normal weight (18.5–24.9 kg/m^2^, reference category), overweight (25–29.9 kg/m^2^), or obese (≥ 30 kg/m^2^). We created a variable identifying people taking psychotropic drugs (i.e., anxiolytics, hypnotics, sedatives, antipsychotics, and antidepressants). The self-reported presence of any pain in the last month before baseline was also considered. Depressive symptoms were assessed through the Montgomery–Åsberg Depression Scale (MADRS) [36], a 10-item subscale driven from the CPRS rating depressive symptom intensity, frequency, and duration. The MADRS scores range between 0 and 60, and scores > 9 reflect the presence of depression [37].

### Statistical analyses

The cross-sectionaly associations between baseline sociodemographic, clinical and lifestyle characteristics and sleep disturbances were examined using the chi-square test for proportions and the Kruskal–Wallis test for continuous variables.

The longitudinal associations between sleep disturbances at baseline and the speed of chronic disease accumulation throughout the 9-year follow-up were analyzed through linear mixed models. We included the interaction term between time and the main exposure as a fixed effect. The *β* coefficients for such interactions can be interpreted as the effect of each level of sleep disturbance on the average annual increase in the number of chronic diseases [29, 30]. Random effects were defined for the intercept and slope, unstructured covariance was assumed, and restricted maximum likelihood estimation was applied. Models were adjusted for all covariates in a cumulative manner: (1) sex, age, and education; (2) additionally by physical activity, smoking, and alcohol consumption; and (3) additionally by the presence of depression (MADRS score > 9), presence of any pain, use of psychotropic medication, and presence of one chronic disease at baseline. Three-way interactions between age, sex, or psychotropic drug use and sleep disturbances × time were tested. To investigate the role of sleep disturbances on the development of specific clusters of chronic diseases [38], we repeated the analyses including only cardiovascular, neuropsychiatric, and musculoskeletal diseases as the outcome in three different models. Last, we explored if specific types of sleep disturbances were associated with multimorbidity development, either among participants reporting any type of sleep disturbances or in the whole study sample.

#### Sensitivity analyses

To evaluate if the associations were driven by any specific chronic conditions either at baseline or at follow-ups, we first compared the distribution of the most prevalent cardiovascular, neuropsychiatric, and musculoskeletal chronic diseases by the presence and severity of sleep disturbances at baseline using the chi-square test. Second, we repeated the analyses concerning cardiovascular, neuropsychiatric, and musculoskeletal diseases after removing the three most common chronic conditions from each group of diseases, one at a time. Third, we repeated the analyses excluding those participants with a diagnosis of dementia or a Mini-Mental State Examination (MMSE) score < 24 at baseline (*n* = 13), to avoid exposure measurement error in these participants. We also repeated the analyses excluding participants with a diagnosis of depression or a MADRS score > 9 at baseline (*n* = 43). All analyses were performed using Stata v.14.

## Results

The mean age of participants was 67.5 years (SD = 7.9) and 58% were female (Supplementary Table 1). Those with missing exposure data (*n* = 28) were more likely to be widow and have an elementary education compared with the study population. Participants with moderate–severe sleep disturbances were more likely to take psychotropic drugs, suffer from depressive symptoms, and self-report pain compared to those with no or mild disturbances (Table 1). There was also a higher proportion of women among participants with moderate–severe disturbances, and this group had higher levels of inadequate physical activity (Table 1). Among the population with sleep disturbances, the three most prevalent types of disturbances were waking up during the night (86.5%), waking up early (51.6%), and problems to fall asleep (39.1%) (Table 2).

**Table 1:** Baseline distribution of sociodemographic, clinical, and lifestyle characteristics by presence and severity of sleep disturbances

	No disturbances, ***n*** = 955	Mild disturbances^**¥**^, ***n*** = 175	Moderate–severe disturbances^**§**^, ***n*** = 59	***p*** value*
**Age,** mean (SD)	67.6 (7.8)	67.9 (8.3)	65.9 (8.1)	0.504
**Sex,** % (*n*)				**0.032**
Female	56.3 (538)	64.0 (112)	69.5 (41)	
Male	43.7 (417)	36.0 (63)	30.5 (18)	
**Education,** % (*n*)				0.332
Elementary	10.4 (99)	8.0 (14)	17.0 (10)	
High school	46.1 (439)	44.6 (78)	39.0 (23)	
University	43.5 (414)	47.4 (83)	44.0 (26)	
**Physical activity,** % (*n*)				**0.001**
Inadequate	18.2 (174)	16.0 (28)	33.9 (20)	
Health-enhancing	50.3 (480)	57.7 (101)	54.2 (32)	
Fitness-enhancing	31.5 (301)	26.3 (46)	11.9 (7)	
**Smoking,** % (*n*)				0.214
Never	43.3 (410)	43.7 (76)	42.4 (25)	
Former	37.7 (358)	43.1 (75)	45.8 (27)	
Current	19.0 (180)	13.2 (23)	11.8 (7)	
**Alcohol,** % (*n*)				0.097
Never	20.6 (195)	20.6 (36)	33.8 (20)	
Light–moderate	61.8 (589)	57.7 (101)	49.2 (29)	
Heavy	17.6 (169)	21.6 (38)	17.0 (10)	
**BMI,** % (*n*)				0.380
Underweight	1.0 (9)	0.0 (0)	1.7 (1)	
Normal weight	45.3 (426)	47.7 (83)	39.0 (23)	
Overweight	42.3 (398)	37.3 (65)	42.3 (25)	
Obesity	11.4 (107)	15.0 (26)	17.0 (10)	
**Psychotropic drugs**^**a**^**,** % (*n*)	7.1 (68)	22.3 (39)	25.4 (15)	**< 0.001**
**Depression**^**b**^**,** % (*n*)	1.1 (10)	5.8 (10)	14.3 (8)	**< 0.001**
**Pain**^**c**^**,** % (*n*)	22.8 (217)	34.5 (60)	47.5 (28)	**< 0.001**

*Obtained through the chi-squared test or the Kruskal–Wallis test, as appropriate

^a^Psychotropic drugs include anxiolytics, hypnotics, sedatives, antipsychotics, and antidepressants

^b^Depression defined according to the Montgomery–Åsberg Depression Rating Scale (MADRS); scores > 9 indicate depression

^c^Self-reported presence of any pain in the last month

^¥^Categories 1–2 “Slight difficulty dropping off to sleep or slightly reduced, light or fitful sleep” of the CPRS question on sleeping problems

^§^Categories 3–4 “Sleep reduced or broken by at least two hours” and 5–6 “Less than two or three hours of sleep” of the CPRS question on sleeping problems

**Table 2:** Type of disturbances among subjects reporting sleep disturbances in the study sample

	Total, ***n*** = 234	< 78 years		≥ 78 years	
		Male, ***n*** = 70	Female, ***n*** = 123	Male, ***n*** = 11	Female, ***n*** = 30
Problems to fall asleep, % (*n*)	39.1 (90)	30.9 (21)	42.5 (51)	45.5 (5)	43.3 (13)
Waking up during night, % (*n*)	86.5 (199)	89.7 (61)	82.6 (100)	90.9 (10)	93.3 (28)
Not being able to fall back asleep, % (*n*)	38.4 (84)	27.7 (18)	43.4 (49)	27.3 (3)	46.7 (14)
Waking up too early, % (*n*)	51.6 (116)	51.5 (34)	52.1 (62)	45.5 (5)	51.7 (15)
Feeling tired > 2 h during the day, % (*n*)	4.4 (10)	6.0 (4)	4.1 (5)	9.1 (1)	100.0 (30)
Taking sleeping drugs, % (*n*)	28.6 (65)	16.4 (11)	30.0 (36)	27.3 (3)	50.0 (15)
Total sleep duration < 6 h*, % (*n*)	27.1 (61)	27.3 (18)	25.0 (30)	27.3 (3)	35.7 (10)

*Defined as short sleep duration by Gildner et al. [9]

**Fig. 1:**
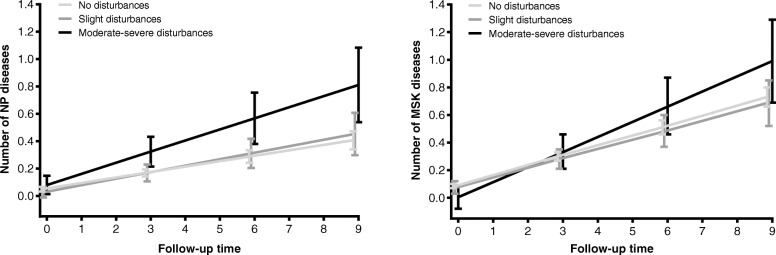
Predicted number of neuropsychiatric (NP) and musculoskeletal (MSK) chronic diseases over the 9-year follow-up in relation to the presence and severity of sleep disturbances at baseline *ß* coefficients for the interaction term between time and the exposure, obtained through linear mixed models. Models adjusted by sex, age, education level, physical activity, smoking, alcohol consumption, BMI, presence of depression (MADRS score > 9) except for the model with NP diseases as the outcome, presence of pain, psychotropic medication, and presence of any chronic disease. Neuropsychiatric diseases: depression and mood diseases, dementia, neurotic or stress-related and somatoform diseases, migraine and facial pain syndromes, peripheral neuropathy, Parkinson or parkinsonism, epilepsy, schizophrenia and delusional diseases, multiple sclerosis, other psychiatric or behavioral diseases, and other neurological diseases. Musculoskeletal diseases: dorsopathies, inflammatory arthropathies, osteoarthritis and other degenerative joint diseases, osteoporosis, and other musculoskeletal and joint diseases.

Moderate–severe sleep disturbances at baseline were associated with a higher speed of chronic disease accumulation throughout the 9-year follow-up, and the direction and magnitude of the association remained stable regardless of potential confounders (Table 3). No significant interactions were found with age, sex, or psychotropic drug use. When focusing on specific groups of chronic diseases, a significant positive association was found between moderate–severe sleep disturbances and neuropsychiatric and musculoskeletal disease accumulation, but not with cardiovascular diseases (Table 4, Fig. 1).

**Table 3:** Association between sleep disturbances at baseline and rate of chronic diseases accumulation throughout the 9-year follow-up

	Model 1		Model 2		Model 3	
	***ß*** (SE)	***p*** value	***ß*** (SE)	***p*** value	***ß*** (SE)	***p*** value
**No disturbances**	*Ref*		*Ref*		*Ref*	
**Mild disturbances**^**¥**^	0.018 (0.032)	0.574	0.019 (0.032)	0.539	0.022 (0.032)	0.496
**Moderate–severe disturbances**^**§**^	0.145 (0.053)	**0.006**	0.147 (0.053)	**0.005**	0.142 (0.054)	**0.008**

*ß* coefficients for the interaction term between time and the exposure, obtained through linear mixed models.

Model 1: adjusted by sex, age, and education level

Model 2: adjusted additionally by physical activity, smoking, alcohol consumption, and BMI

Model 3: adjusted additionally by the presence of depression (MADRS score > 9), presence of any pain, psychotropic medication, and presence of one chronic disease at baseline

^¥^Categories 1–2 “Slight difficulty dropping off to sleep or slightly reduced, light or fitful sleep” of the CPRS question on sleeping problems

^§^Categories 3–4 “Sleep reduced or broken by at least two hours” and 5–6 “Less than two or three hours of sleep” of the CPRS question on sleeping problems

**Table 4:** Association between sleep disturbances at baseline and rate of cardiovascular (CV), neuropsychiatric (NP), and musculoskeletal (MSK) chronic disease accumulation throughout the 9-year follow-up

	CV diseases		NP diseases		MSK diseases	
	***ß*** (SE)	***p*** value	***ß*** (SE)	***p*** value	***ß*** (SE)	***p*** value
**No disturbances**	*Ref*		*Ref*		*Ref*	
**Mild disturbances**^**¥**^	− 0.012 (0.011)	0.267	0.008 (0.009)	0.419	− 0.003 (0.010)	0.735
**Moderate–severe disturbances**^**§**^	0.001 (0.018)	0.995	0.041 (0.016)	**0.008**	0.038 (0.017)	**0.025**

*ß* coefficients for the interaction term between time and the exposure, obtained through linear mixed models. Models adjusted by sex, age, education level, physical activity, smoking, alcohol consumption, BMI, presence of depression (MADRS score > 9) except for the model with NP diseases as the outcome, presence of pain, psychotropic medication, and presence of one chronic disease at baseline. Cardiovascular diseases: ischemic heart disease, heart failure, atrial fibrillation, cerebrovascular disease, cardiac valve diseases, bradycardias or conduction diseases, peripheral vascular disease, and other cardiovascular diseases. Neuropsychiatric diseases: depression and mood diseases, dementia, neurotic or stress-related and somatoform diseases, migraine and facial pain syndromes, peripheral neuropathy, Parkinson or parkinsonism, epilepsy, schizophrenia and delusional diseases, multiple sclerosis, other psychiatric or behavioral diseases, and other neurological diseases. Musculoskeletal diseases: dorsopathies, inflammatory arthropathies, osteoarthritis and other degenerative joint diseases, osteoporosis, and other musculoskeletal and joint diseases

^¥^Categories 1–2 “Slight difficulty dropping off to sleep or slightly reduced, light or fitful sleep” of the CPRS question on sleeping problems

^§^Categories 3–4 “Sleep reduced or broken by at least two hours” and 5–6 “Less than two or three hours of sleep” of the CPRS question on sleeping problems

When focusing on specific types of sleep disturbances, “feeling tired more than 2 hours during the day” was consistently associated with the rate of total, neuropsychiatric, and musculoskeletal disease accumulation (Supplementary Tables 2A and B).

Except for ischemic heart disease and depression and mood diseases (borderline statistical significance), no other differences were found in the baseline distribution of the most prevalent cardiovascular, neuropsychiatric, and musculoskeletal chronic diseases by presence and severity of sleep disturbances (Supplementary Table 3). When the analyses in Table 4 were repeated after removing one condition at a time (only the individual condition specified) from each group of chronic diseases (Supplementary Table 4), similar findings were obtained. The one exception was musculoskeletal disease; when osteoarthritis and other degenerative joint diseases were removed, the association was no longer significant. Results remained the same when participants with a baseline diagnosis of dementia or MMSE < 24 were excluded from the study sample (Supplementary Table 5). When participants with depression and mood diseases or a MADRS score > 9 were excluded, the direction of the association between moderate–severe sleep disturbances and the rate of chronic disease accumulation remained the same, even if it did not reach statistical significance (Supplementary Table 6).

## Discussion

This study sought to investigate the association between sleep disturbances and the speed of multimorbidity development in older adults. Results showed that moderate–severe sleep disturbances at baseline were independently associated with a higher speed of chronic disease accumulation during the 9-year follow-up. With regard to specific groups of chronic diseases, a significant positive association was found between moderate–severe sleep disturbances and neuropsychiatric and musculoskeletal disease accumulation, but not with cardiovascular diseases. To the best of our knowledge, this is the first study to longitudinally investigate sleep and speed of multimorbidity development after a long follow-up duration in a large population-based study. Considering that this is the first study to show such associations, direct comparisons cannot be made with previous literature.

Our finding on the association between sleep disturbances and accumulation of neuropsychiatric diseases supports previous cross-sectional results from nationally representative samples, where of several conditions, depression was the only one consistently associated with sleep disturbances [25]. In a Korean study, baseline insomnia was associated with incident depression after two years [28]. In our study, even when depression was removed from the analyses, the associations remained robust. This may highlight the additional strong associations between sleep disturbances and dementia and migraine and facial pain syndromes. Our findings are consistent with studies showing that sleep disturbances are associated with dementia risk [10, 11]. This study additionally supports a recent meta-analysis showing that obstructive sleep apnea is associated with cerebrovascular diseases [39]. The current findings provide further evidence regarding the association between sleep disturbances and incidence of neuropsychiatric conditions, but also a faster accumulation of neuropsychiatric conditions.

Regarding our finding on the association between sleep disturbances and speed of musculoskeletal disease accumulation, when osteoarthritis and other degenerative joint diseases were removed from the analyses, the associations were no longer significant, suggesting a central role of these conditions. A recent systematic review and meta-analysis has shown that having osteoarthritis was associated with the presence of other chronic conditions and multimorbidity in a dose-dependent manner [40]. Our results add to this literature by highlighting the role of osteoarthritis and degenerative joint disease in the associations between sleep disturbances and musculoskeletal disease accumulation. These findings are also consistent with cross-sectional results from Brazil demonstrating a U-shaped association; both short sleep and long sleep duration were associated with rheumatism/arthritis/arthrosis and osteoporosis [23]. Short sleep duration was also associated with back pain problems. Similarly, in the aforementioned multi-national study, sleep disturbances were associated with arthritis [25]. Another cross-sectional study among older adults in Germany showed that insomnia and daytime tiredness were associated with joint diseases [27]. Increased pain and inflammation are proposed as central mechanisms in these associations, and fatigue may also play a role [41, 42].

In the current study, although the lack of association between sleep disturbances and cardiovascular disease accumulation may appear to contradict previous findings, comparisons are in fact non-feasible, considering the cross-sectional study designs, differences in age groups, definitions of multimorbidity, sleep disturbance measurements, and non-included potential confounders (e.g., BMI, hypnotics, education, smoking, alcohol consumption, exercise) in the previous studies reporting positive associations [9, 23, 25, 27]. These methodological differences may contribute to the observed differences. Moreover, in our study, the non-significant association between sleep disturbances and cardiovascular disease may reflect a selection bias; in this sample of older adults, those with cardiovascular diseases may have not participated in this study or its follow-up assessments due to earlier adverse outcomes (e.g., mortality and disability). Also, participants with cardiovascular diseases tend to have other coexisting chronic conditions [43] and may have been excluded from our study sample, which may have prevented us from finding such an association.

The results showed that there were no significant interactions between sleep disturbances and age or sex. A previous study did find sex-specific associations; insomnia and specific insomnia symptoms were associated with multimorbidity among women, but not among men [27]. This study differed from the current one as it was cross-sectional and it did not adjust for depression, chronic diseases, and pain. Also, the sample was slightly older in age with lower education levels. Considering the previously reported sex differences in sleep, this topic warrants further investigation among older adults [44].

Several mechanisms may underlie the associations observed. First, an important mechanism underlying neuropsychiatric and cardiovascular conditions is dysregulations in the hypothalamic–pituitary–adrenal (HPA) axis [45, 46], which are also implicated in sleep disturbances. Poor sleep quality enhances the reactivity of the HPA axis and is associated with higher daytime cortisol levels [47, 48], and altered cortisol awakening response [49]. The relationship between sleep disturbances and HPA axis dysregulations may be bidirectional, as HPA axis hyperactivation is also associated with poor sleep [50]. Second, sleep disturbances are connected with dysregulations in inflammatory markers. For example, sleep disturbances are associated with higher levels of C-reactive protein (CRP) and interleukin-6 (IL-6) [51], which are also commonly elevated in musculoskeletal and neuropsychiatric diseases [52, 53], and among people with a fast accumulation of chronic diseases [54]. These mechanisms may also impact serotonin, a neurotransmitter that plays a role in regulating sleep-wake states, and also regulates mood and tends to be depleted in depression, representing an important component in the pathophysiology of the disorder [55–57]. Another potential mechanism may be through the neuropeptide orexin, which regulates sleep-wake cycles and the circadian rhythm and plays a role in cognitive function and the cardiovascular system [58]. Orexin receptor antagonists have been investigated for their potential therapeutic effects for the treatment of insomnia [59]. Sleep disturbances are also associated with compromises in metabolic health and can increase the risk for obesity, dyslipidemia, hypertension, and impaired glucose metabolism, all of which increase the risk for cardiovascular conditions [60]. Lifestyle factors, beyond those taken into account in our models (e.g., changes in appetite, diet, and polypharmacy), which also increase cardiometabolic risk and degrade sleep quality [60–62].

This study has a few limitations. The measure of sleep was self-reported and measured at a single time point. Objective measures of chronic sleep disturbances may provide more reliable data. While the current study investigates sleep in late life and its association with multimorbidity, evidence from other studies highlights the importance of midlife sleep and adverse outcomes [11, 14, 63]. Regarding the feelings of tiredness during the day, we could not examine the source of tiredness, as this additional information was not collected. We also did not have clinical measures of sleep apnea, which is associated with multimorbidity [64, 65], and some parameters such as sleep efficiency and sleep latency are not included. The external validity of the study is limited because the sample only included healthy participants from SNAC-K population, which is wealthier than average in Sweden, and may have better lifestyle and fewer vascular risk factors (e.g., obesity). Attrition due to mortality and dropout might have affected the results. Although we excluded subjects with multimorbidity at baseline, and adjusted our models for several confounders, the possibility of reverse causality or residual confounding cannot be fully discarded given the potential heterogeneity in subclinical health states leading to multimorbidity. Finally, sleep disturbances may in fact represent the symptoms of already established disorders (e.g., depression, arthritis). These could be the single disorders that people with only one disease suffer from (who were not excluded from the study sample) or disorders that were undiagnosed yet at baseline. In both cases, this could potentially lead to reverse causality regarding the associations between sleep disturbances and multimorbidity.

Despite the limitations, this study has several strengths. First, this study is population-based, longitudinal, with baseline measures of sleep disturbances in old age, and a 9-year follow-up. Second, chronic conditions were either objectively measured by physicians within the SNAC-K study or retrieved in medical health records, including a wide range of diseases. Third, this study provides data on the speed of multimorbidity accumulation, a proxy for the speed of biological aging [66]. Fourth, individuals with multimorbidity at baseline were excluded from analyses, which limits the risk of reverse causality. Fifth, sleep was measured using a validated scale including several parameters of sleep disturbances. Finally, we have adjusted for potential confounders including medications.

Sleep disturbances and circadian rhythm disruptions may be a proxy of biological vulnerability or early disease stage [67] and therefore may help identify individuals with potentially worse multimorbidity trajectories. Sleep disturbances tend to be chronic, and interventions need to target a comprehensive range of behavioral/lifestyle factors that impact sleep. Evidence from randomized controlled trials offers support for cognitive behavioral therapy for insomnia (CBT-I) as an effective and comprehensive non-pharmacological approach [61, 62, 68]. Considering the potential side effects of pharmacological treatments, they are recommended as a last resort [61].

## Conclusion

In conclusion, for the first time, this study showed that moderate–severe sleep disturbances were associated with a higher speed of overall, neuropsychiatric and musculoskeletal chronic disease accumulation over time.

## Supplementary Information

**Additional file 1:** **Supplementary Table 1.** Baseline sociodemographic, clinical and lifestyle characteristics of the study population by age and sex. **Supplementary Table 2.** Association between type of sleep disturbance at baseline and rate of total and specific groups of chronic disease accumulation throughout the nine-year follow-up. **Supplementary Table 3.** Baseline distribution of most prevalent cardiovascular^a^ (CV), neuropsychiatric^b^ (NP) and musculoskeletal^c^ (MSK) chronic diseases by presence and severity of sleep disturbances. **Supplementary Table 4.** Association between presence and severity of sleep disturbances at baseline and rate of cardiovascular (CV), neuropsychiatric (NP) and musculoskeletal (MSK) chronic disease accumulation throughout the nine-year follow-up. Analyses performed after removing most prevalent chronic conditions, one at a time, from each group of chronic diseases. **Supplementary Table 5.** Association between presence and severity of sleep disturbances at baseline and rate of total and specific groups of chronic disease accumulation throughout the nine-year follow-up. Analyses excluding participants with dementia or cognitive impairment at baseline. **Supplementary Table 6.** Association between presence and severity of sleep disturbances at baseline and rate of total and specific groups of chronic disease accumulation throughout the nine-year follow-up. Analyses excluding participants with depression at baseline*.

## Data Availability

Data are from the SNAC-K project, a population-based study on aging and dementia (http://www.snac-k.se/). Access to these original data is available to the research community upon approval by the SNAC-K data management and maintenance committee. Applications for accessing these data can be submitted to Maria Wahlberg (Maria.Wahlberg@ki.se) at the Aging Research Center, Karolinska Institutet, Stockholm, Sweden.
