# Supplementary material for: Sleep disturbances and the speed of multimorbidity development in old age: results from a longitudinal population-based study
Source: BMC Med. 2020 Dec 7;18:382. doi: 10.1186/s12916-020-01846-w (PMC7720467; doi:10.1186/s12916-020-01846-w)
Supplement: Supplementary file 1 — Additional file 1: Supplementary Table 1. Baseline sociodemographic, clinical and lifestyle characteristics of the study population by age and sex. Supplementary Table 2. Association between type of sleep disturbance at baseline and rate of total and specific groups of chronic disease accumulation throughout the nine-year follow-up. Supplementary Table 3. Baseline distribution of most prevalent cardiovasculara (CV), neuropsychiatricb (NP) and musculoskeletalc (MSK) chronic diseases by presence and severity of sleep disturbances. Supplementary Table 4. Association between presence and severity of sleep disturbances at baseline and rate of cardiovascular (CV), neuropsychiatric (NP) and musculoskeletal (MSK) chronic disease accumulation throughout the nine-year follow-up. Analyses performed after removing most prevalent chronic conditions, one at a time, from each group of chronic diseases. Supplementary Table 5. Association between presence and severity of sleep disturbances at baseline and rate of total and specific groups of chronic disease accumulation throughout the nine-year follow-up. Analyses excluding participants with dementia or cognitive impairment at baseline. Supplementary Table 6. Association between presence and severity of sleep disturbances at baseline and rate of total and specific groups of chronic disease accumulation throughout the nine-year follow-up. Analyses excluding participants with depression at baseline*. [file 12916_2020_1846_MOESM1_ESM.docx]

**Supplementary Table 1. Baseline sociodemographic, clinical and lifestyle characteristics of the study population by age and sex.**

|  | **Total**  **N=1189** | **<78 years** | | **≥78 years** | |
| --- | --- | --- | --- | --- | --- |
|  |  | **Male**  **n=429** | **Female**  **n=548** | **Male**  **n=69** | **Female**  **n=141** |
| **Education,** % (n)  Elementary  High school  University | 10.4 (124)  45.5 (540)  44.1 (523) | 7.7 (33)  40.8 (175)  51.5 (221) | 7.3 (40)  47.8 (262)  44.9 (246) | 21.7 (15)  46.4 (32)  31.9 (22) | 25.0 (35)  50.7 (71)  24.3 (34) |
| **Civil status,** % (n)  Married  Widow  Divorced  Unmarried | 56.8 (673)  12.4 (147)  14.2 (168)  16.7 (198) | 71.3 (306)  2.8 (12)  9.6 (41)  16.3 (70) | 51.6 (282)  10.6 (58)  19.7 (108)  18.1 (99) | 68.1 (47)  15.9 (11)  4.4 (3)  11.6 (8) | 27.0 (38)  46.8 (66)  11.4 (16)  14.8 (21) |
| **Living place,** % (n)  Community  Nursing home | 99.8 (1186)  0.2 (3) | 100.0 (429)  0.0 (0) | 99.8 (547)  0.2 (1) | 100.0 (69)  0.0 (0) | 98.6 (139)  1.4 (2) |
| **Physical activity,** % (n)  Inadequate  Health enhancing  Fitness enhancing | 18.7 (222)  51.6 (613)  29.7 (354) | 20.5 (88)  47.3 (203)  32.2 (138) | 15.9 (87)  52.7 (289)  31.4 (172) | 17.4 (12)  53.6 (37)  29.0 (20) | 24.1 (34)  58.9 (83)  17.0 (24) |
| **Smoking,** % (n)  Never  Former  Current | 43.3 (511)  39.0 (460)  17.7 (210) | 34.7 (148)  46.3 (198)  19.0 (81) | 44.7 (244)  36.3 (198)  19.1 (104) | 37.7 (26)  49.3 (34)  13.0 (9) | 66.9 (93)  21.6 (30)  11.5 (16) |
| **Alcohol,** % (n)  Never  Light-moderate  Heavy | 21.2 (251)  60.6 (719)  18.2 (217) | 13.3 (57)  75.4 (324)  11.2 (48) | 18.8 (103)  54.4 (298)  26.8 (147) | 29.0 (20)  68.1 (47)  2.9 (2) | 50.4 (71)  35.5 (50)  14.2 (20) |
| **BMI,** % (n)  Underweight  Normal weight  Overweight  Obesity | 0.9 (10)  45.4 (532)  41.6 (488)  12.1 (143) | 0.2 (1)  37.4 (159)  50.0 (213)  12.4 (53) | 0.4 (2)  51.3 (280)  35.9 (196)  12.5 (68) | 1.5 (1)  43.5 (30)  46.4 (32)  8.7 (6) | 4.6 (6)  47.7 (63)  35.6 (47)  12.1 (16) |
| **Psychotropic drugs ^a^,** % (n) | 10.3 (122) | 4.4 (19) | 10.6 (58) | 14.5 (10) | 24.1 (34) |
| **Depression ^b^,** %(n) | 2.4 (28) | 1.9 (8) | 2.8 (15) | 1.5 (1) | 2.9 (4) |
| **Pain ^c^,** % (n) | 25.7 (305) | 17.5 (75) | 33.7 (184) | 18.8 (13) | 23.6 (33) |

^a^ Psychotropic drugs include: anxiolytics, hypnotics, sedatives, antipsychotics and antidepressants.

^b^ Depression defined according to the Montgomery–Åsberg Depression Rating Scale (MADRS); scores >9 indicate depression.

^c^ Self-reported presence of any pain in the last month.

**Supplementary Table 2. Association between type of sleep disturbance at baseline and rate of total and specific groups of chronic disease accumulation throughout the nine-year follow-up.**

1. **Analyses including subjects reporting sleep disturbances in the study sample.**

|  | **All chronic diseases** | | **CV diseases** | | **NP diseases** | | **MSK diseases** | |
| --- | --- | --- | --- | --- | --- | --- | --- | --- |
|  | **ß (SE)** | **p-value** | **ß (SE)** | **p-value** | **ß (SE)** | **p-value** | **ß (SE)** | **p-value** |
| **Problems to fall asleep** | 0.066 (0.053) | 0.216 | -0.007 (0.016) | 0.689 | 0.023 (0.018) | 0.211 | -0.018 (0.018) | 0.323 |
| **Waking up during night** | 0.207  (0.072) | **0.004** | 0.038 (0.023) | 0.095 | 0.009 (0.025) | 0.717 | -0.008 (0.025) | 0.761 |
| **Not being able to fall back asleep** | -0.0.16 (0.054) | 0.768 | -0.023 (0.017) | 0.175 | 0.005 (0.019) | 0.806 | 0.034 (0.018) | 0.056 |
| **Waking up too early** | -0.020 (0.052) | 0.699 | 0.009 (0.016) | 0.592 | 0.004 (0.018) | 0.841 | 0.027 (0.017) | 0.113 |
| **Feeling tired >2 hours during the day** | 0.203 (0.112) | 0.070 | 0.006 (0.035) | 0.875 | 0.089 (0.039) | **0.021** | 0.089 (0.038) | **0.018** |
| **Taking sleeping drugs** | 0.040 (0.060) | 0.506 | -0.037 (0.018) | **0.047** | 0.022 (0.020) | 0.288 | 0.026 (0.020) | 0.202 |
| **Total sleep duration <6 hours*** | 0.031 (0.059) | 0.601 | 0.020 (0.019) | 0.276 | 0.025 (0.020) | 0.214 | -0.024 (0.020) | 0.242 |

1. **Analyses including all the study sample.**

|  | **All chronic diseases** | | **CV diseases** | | **NP diseases** | | **MSK diseases** | |
| --- | --- | --- | --- | --- | --- | --- | --- | --- |
|  | **ß (SE)** | **p-value** | **ß (SE)** | **p-value** | **ß (SE)** | **p-value** | **ß (SE)** | **p-value** |
| **Problems to fall asleep** | 0.095 (0.044) | **0.032** | -0.013 (0.015) | 0.392 | 0.031 (0.013) | **0.020** | -0.008 (0.014) | 0.589 |
| **Waking up during night** | 0.093  (0.030) | **0.002** | -0.001 (0.010) | 0.898 | 0.018 (0.009) | **0.042** | 0.006 (0.010) | 0.554 |
| **Not being able to fall back asleep** | 0.058 (0.044) | 0.189 | -0.020 (0.015) | 0.194 | 0.021 (0.013) | 0.110 | 0.027 (0.014) | **0.049** |
| **Waking up too early** | 0.039 (0.039) | 0.322 | -0.002 (0.013) | 0.857 | 0.018 (0.011) | 0.109 | 0.020 (0.012) | 0.108 |
| **Feeling tired >2 hours during the day** | 0.240 (0.112) | **0.032** | -0.001 (0.038) | 0.993 | 0.100 (0.033) | **0.002** | 0.009 (0.035) | **0.010** |
| **Taking sleeping drugs** | 0.081 (0.054) | 0.133 | -0.037 (0.018) | **0.044** | 0.030 (0.016) | 0.059 | 0.026 (0.017) | 0.121 |
| **Total sleep duration <6 hours*** | 0.078 (0.053) | 0.145 | 0.011 (0.018) | 0.552 | 0.032 (0.016) | **0.040** | -0.011 (0.017) | 0.521 |

ß coefficients for the interaction term between time and the exposure, obtained through linear mixed models.

Models adjusted by sex, age, education level, physical activity, smoking, alcohol consumption, BMI, presence of depression (MADRS score >9) except for the model with NP diseases as the outcome, presence of pain, psychotropic medication and presence of any chronic disease.

^a^ Cardiovascular diseases: ischemic heart disease, heart failure, atrial fibrillation, cerebrovascular disease, cardiac valve diseases, bradycardias or conduction diseases, peripheral vascular disease, other cardiovascular diseases.

^b^ Neuropsychiatric diseases: depression and mood diseases, dementia, neurotic or stress-related and somatoform diseases, migraine and facial pain syndromes, peripheral neuropathy, Parkinson or parkinsonism, epilepsy, schizophrenia and delusional diseases, multiple sclerosis, other psychiatric or behavioral diseases, other neurological diseases.

^c^ Musculoskeletal diseases: dorsopathies, inflammatory arthropathies, osteoarthritis and other degenerative joint diseases, osteoporosis, other musculoskeletal and joint diseases.

^*^ Defined as short sleep duration by Gildner TE, et al. [9].

**Supplementary Table 3. Baseline distribution of most prevalent cardiovascular**^a^ **(CV), neuropsychiatric**^b^ **(NP) and musculoskeletal**^c^ **(MSK) chronic diseases by presence and severity of sleep disturbances.**

|  | **No disturbances**  **n=955** | **Mild**  **disturbances^¥^**  **n=175** | **Moderate-severe disturbances^§^**  **n=59** | **p-value^*^** |
| --- | --- | --- | --- | --- |
| **CV diseases,** % (n) |  |  |  |  |
| Ischemic heart disease | 1.4 (13) | 6.3 (11) | 1.7 (1) | **<0.001** |
| Atrial fibrillation | 0.8 (8) | 1.7 (3) | 1.7 (1) | 0.489 |
| Cerebrovascular disease | 1.5 (14) | 0.6 (1) | 1.7 (1) | 0.622 |
| **NP diseases,** % (n) |  |  |  |  |
| Depression and mood diseases | 1.5 (14) | 2.9 (5) | 5.2 (3) | 0.076 |
| Dementia | 0.8 (8) | 0.0 (0) | 0.0 (0) | 0.373 |
| Migraine and facial pain syndromes | 1.2 (11) | 2.3 (4) | 1.7 (1) | 0.475 |
| **MSK diseases,** % (n) |  |  |  |  |
| Dorsopathies | 1.7 (16) | 1.1 (2) | 0.0 (0) | 0.539 |
| Osteoarthritis and other degenerative joint diseases | 3.6 (34) | 5.1 (9) | 1.7 (1) | 0.519 |
| Inflammatory arthropathies | 0.8 (8) | 0.6 (1) | 0.0 (0) | 0.736 |

^*^ Obtained through Chi-squared test.

^a^ Cardiovascular diseases: ischemic heart disease, heart failure, atrial fibrillation, cerebrovascular disease, cardiac valve diseases, bradycardias or conduction diseases, peripheral vascular disease, other cardiovascular diseases.

^b^ Neuropsychiatric diseases: depression and mood diseases, dementia, neurotic or stress-related and somatoform diseases, migraine and facial pain syndromes, peripheral neuropathy, Parkinson or parkinsonism, epilepsy, schizophrenia and delusional diseases, multiple sclerosis, other psychiatric or behavioral diseases, other neurological diseases.

^c^ Musculoskeletal diseases: dorsopathies, inflammatory arthropathies, osteoarthritis and other degenerative joint diseases, osteoporosis, other musculoskeletal and joint diseases.

**^¥^** Categories 1-2 “Slight difficulty dropping off to sleep or slightly reduced, light or fitful sleep” of the CPRS question on sleeping problems.

**^§^** Categories 3-4 “Sleep reduced or broken by at least two hours” and 5-6 “Less than two or three hours of sleep” of the CPRS question on sleeping problems.

**Supplementary Table 4. Association between presence and severity of sleep disturbances at baseline and rate of cardiovascular (CV), neuropsychiatric (NP) and musculoskeletal (MSK) chronic disease accumulation throughout the nine-year follow-up. Analyses performed after removing most prevalent chronic conditions, one at a time, from each group of chronic diseases.**

|  | **Mild^¥^**  **vs no disturbances** | | **Moderate-severe^§^**  **vs no disturbances** | |
| --- | --- | --- | --- | --- |
|  | **ß (SE)** | **p-value** | **ß (SE)** | **p-value** |
| **Cardiovascular diseases**^a^ |  |  |  |  |
| Overall estimation | -0.012 (0.011) | 0.267 | 0.001 (0.018) | 0.995 |
| Ischemic heart disease removed | -0.010 (0.010) | 0.303 | -0.004 (0.016) | 0.803 |
| Atrial fibrillation removed | -0.006 (0.010) | 0.545 | 0.005 (0.016) | 0.758 |
| Cerebrovascular disease removed | -0.012 (0.010) | 0.234 | -0.001 (0.017) | 0.992 |
| **Neuropsychiatric diseases**^b^ | | | | |
| Overall estimation | 0.008 (0.009) | 0.419 | 0.041 (0.016) | **0.008** |
| Depression and mood diseases removed | 0.004 (0.008) | 0.594 | 0.034 (0.013) | **0.011** |
| Dementia removed | 0.008 (0.008) | 0.285 | 0.037 (0.013) | **0.004** |
| Migraine and facial pain syndromes | 0.008 (0.009) | 0.385 | 0.037 (0.014) | **0.012** |
| **Musculoskeletal diseases**^c^ | | | | |
| Overall estimation | -0.003 (0.010) | 0.735 | 0.038 (0.017) | **0.025** |
| Dorsopathies removed | 0.001 (0.009) | 0.985 | 0.040 (0.016) | **0.010** |
| Osteoarthritis and other degenerative joint diseases removed | -0.002 (0.007) | 0.770 | 0.017 (0.012) | 0.174 |
| Inflammatory arthropathies removed | -0.001 (0.010) | 0.946 | 0.042 (0.017) | **0.011** |

ß coefficients for the interaction term between time and the exposure, obtained through linear mixed models.

Models adjusted by sex, age, education level, physical activity, smoking, alcohol consumption, BMI, presence of depression (MADRS score >9) except for the model with NP diseases as the outcome, presence of pain, psychotropic medication and presence of any chronic disease.

^a^ Cardiovascular diseases: ischemic heart disease, heart failure, atrial fibrillation, cerebrovascular disease, cardiac valve diseases, bradycardias or conduction diseases, peripheral vascular disease, other cardiovascular diseases.

^b^ Neuropsychiatric diseases: depression and mood diseases, dementia, neurotic or stress-related and somatoform diseases, migraine and facial pain syndromes, peripheral neuropathy, Parkinson or parkinsonism, epilepsy, schizophrenia and delusional diseases, multiple sclerosis, other psychiatric or behavioral diseases, other neurological diseases.

^c^ Musculoskeletal diseases: dorsopathies, inflammatory arthropathies, osteoarthritis and other degenerative joint diseases, osteoporosis, other musculoskeletal and joint diseases.

**^¥^** Categories 1-2 “Slight difficulty dropping off to sleep or slightly reduced, light or fitful sleep”.

**^§^** Categories 3-4 “Sleep reduced or broken by at least two hours” and 5-6 “Less than two or three hours of sleep”.

**Supplementary Table 5. Association between presence and severity of sleep disturbances at baseline and rate of total and specific groups of chronic disease accumulation throughout the nine-year follow-up. Analyses excluding participants with dementia or cognitive impairment at baseline.**

|  | **All chronic diseases** | | **CV diseases** | | **NP diseases** | | **MSK diseases** | |
| --- | --- | --- | --- | --- | --- | --- | --- | --- |
|  | **ß (SE)** | **p-value** | **ß (SE)** | **p-value** | **ß (SE)** | **p-value** | **ß (SE)** | **p-value** |
| **No disturbances** | *ref* |  | *ref* |  | *ref* |  | *ref* |  |
| **Mild disturbances^¥^** | 0.025 (0.031) | 0.430 | -0.012 (0.011) | 0.287 | 0.008 (0.009) | 0.388 | -0.003 (0.010) | 0.734 |
| **Moderate-severe disturbances^§^** | 0.126 (0.053) | **0.019** | 0.002 (0.019) | 0.909 | 0.030 (0.016) | 0.057 | 0.037 (0.017) | **0.032** |

ß coefficients for the interaction term between time and the exposure, obtained through linear mixed models.

Models adjusted by sex, age, education level, physical activity, smoking, alcohol consumption, BMI, presence of depression (MADRS score >9) except for the model with NP diseases as the outcome, presence of pain, psychotropic medication and presence of any chronic disease.

^a^ Cardiovascular diseases: ischemic heart disease, heart failure, atrial fibrillation, cerebrovascular disease, cardiac valve diseases, bradycardias or conduction diseases, peripheral vascular disease, other cardiovascular diseases.

^b^ Neuropsychiatric diseases: depression and mood diseases, dementia, neurotic or stress-related and somatoform diseases, migraine and facial pain syndromes, peripheral neuropathy, Parkinson or parkinsonism, epilepsy, schizophrenia and delusional diseases, multiple sclerosis, other psychiatric or behavioral diseases, other neurological diseases.

^c^ Musculoskeletal diseases: dorsopathies, inflammatory arthropathies, osteoarthritis and other degenerative joint diseases, osteoporosis, other musculoskeletal and joint diseases.

**^¥^** Categories 1-2 “Slight difficulty dropping off to sleep or slightly reduced, light or fitful sleep” of the CPRS question on sleeping problems.

**^§^** Categories 3-4 “Sleep reduced or broken by at least two hours” and 5-6 “Less than two or three hours of sleep” of the CPRS question on sleeping problems.

**Supplementary Table 6. Association between presence and severity of sleep disturbances at baseline and rate of total and specific groups of chronic disease accumulation throughout the nine-year follow-up. Analyses excluding participants with depression at baseline*.**

|  | **All chronic diseases** | | **CV diseases** | | **NP diseases** | | **MSK diseases** | |
| --- | --- | --- | --- | --- | --- | --- | --- | --- |
|  | **ß (SE)** | **p-value** | **ß (SE)** | **p-value** | **ß (SE)** | **p-value** | **ß (SE)** | **p-value** |
| **No disturbances** | *ref* | *ref* | *ref* |  | *ref* |  | *ref* |  |
| **Mild disturbances^¥^** | 0.012  (0.033) | 0.703 | -0.012 (0.011) | 0.277 | 0.003 (0.009) | 0.721 | -0.004 (0.010) | 0.673 |
| **Moderate-severe disturbances^§^** | 0.101 (0.057) | 0.074 | 0.004 (0.020) | 0.858 | 0.014 (0.016) | 0.399 | 0.037 (0.018) | **0.041** |

* Participants had either the chronic disease category “depression and mood diseases” or a MADRS score >9.

ß coefficients for the interaction term between time and the exposure, obtained through linear mixed models.

Models adjusted by sex, age, education level, physical activity, smoking, alcohol consumption, BMI, presence of pain, psychotropic medication and presence of any chronic disease.

^a^ Cardiovascular diseases: ischemic heart disease, heart failure, atrial fibrillation, cerebrovascular disease, cardiac valve diseases, bradycardias or conduction diseases, peripheral vascular disease, other cardiovascular diseases.

^b^ Neuropsychiatric diseases: depression and mood diseases, dementia, neurotic or stress-related and somatoform diseases, migraine and facial pain syndromes, peripheral neuropathy, Parkinson or parkinsonism, epilepsy, schizophrenia and delusional diseases, multiple sclerosis, other psychiatric or behavioral diseases, other neurological diseases.

^c^ Musculoskeletal diseases: dorsopathies, inflammatory arthropathies, osteoarthritis and other degenerative joint diseases, osteoporosis, other musculoskeletal and joint diseases.

**^¥^** Categories 1-2 “Slight difficulty dropping off to sleep or slightly reduced, light or fitful sleep” of the CPRS question on sleeping problems.

**^§^** Categories 3-4 “Sleep reduced or broken by at least two hours” and 5-6 “Less than two or three hours of sleep” of the CPRS question on sleeping problems.
